# The Natural Product Secoemestrin C Inhibits Colorectal Cancer Stem Cells via p38–S100A8 Feed-Forward Regulatory Loop

**DOI:** 10.3390/cells13070620

**Published:** 2024-04-03

**Authors:** Huimin Zhou, Minghua Chen, Cong Zhao, Rongguang Shao, Yanni Xu, Wuli Zhao

**Affiliations:** 1 State Key Laboratory of Respiratory Health and Multimorbidity, Key Laboratory of Antibiotic Bioengineering, Ministry of Health, Laboratory of Oncology, Institute of Medicinal Biotechnology, Chinese Academy of Medical Sciences & Peking Union Medical College, Beijing 100050, China; zhouhuimin@imb.pumc.edu.cn (H.Z.); zhaocong@imb.pumc.edu.cn (C.Z.); shaor@imb.pumc.edu.cn (R.S.); 2 NHC Key Laboratory of Biotechnology of Antibiotics, National Center for New Microbial Drug Screening, Institute of Medicinal Biotechnology, Chinese Academy of Medical Sciences & Peking Union Medical College, 1 Tiantan Xili, Beijing 100050, China; mingsunlight@sina.com

**Keywords:** colorectal cancer, CSCs, p38, S100A8, secoemestrin C, stemness

## Abstract

Cancer stem cells (CSCs) are closely associated with tumor initiation, metastasis, chemoresistance, and recurrence, which represent some of the primary obstacles to cancer treatment. Targeting CSCs has become an important therapeutic approach to cancer care. Secoemestrin C (Sec C) is a natural compound with strong anti-tumor activity and low toxicity. Here, we report that Sec C effectively inhibited colorectal CSCs and non-CSCs concurrently, mainly by inhibiting proliferation, self-renewal, metastasis, and drug resistance. Mechanistically, RNA-seq analysis showed that the pro-inflammation pathway of the IL17 axis was enriched, and its effector S100A8 was dramatically decreased in Sec C-treated cells, whose roles in the stemness of CSCs have not been fully clarified. We found that the overexpression of S100A8 hindered the anti-CSCs effect of Sec C, and S100A8 deficiency attenuated the stemness traits of CSCs to enhance the Sec C killing activity on them. Meanwhile, the p38 signal pathway, belonging to the IL17 downstream axis, can also mediate CSCs and counter with Sec C. Notably, we found that S100A8 upregulation increased the p38 protein level, and p38, in turn, promoted S100A8 expression. This indicated that p38 may have a mutual feedback loop with S100A8. Our study discovered that Sec C was a powerful anti-colorectal CSC agent, and that the positive feedback loop of p38–S100A8 mediated Sec C activity. This showed that Sec C could act as a promising clinical candidate in colorectal cancer treatment, and S100A8 could be a prospective drug target.

## 1. Introduction

Colorectal cancer is the world’s third most common malignant tumor and the second main reason for cancer death, simultaneously being the second and third most familiar cancer among women and men, with over 1.8 million confirmed cases annually [1,2]. Among these populations, 20% suffer from metastatic colorectal cancer, and 30–40% will relapse after receiving surgery [3,4]. Moreover, patients with colorectal cancer have a poor prognosis, with a 5-year survival rate of 65% and a 10-year survival rate of 58% [5]. Usually, obesity, smoking, eating disorders, and alcohol abuse are key factors leading to colorectal cancer, which is always accompanied by symptoms such as intestinal bleeding, pain, weight loss, and spasms [5,6,7]. At present, the clinical treatment methods for colorectal cancer mainly include chemotherapy, radiotherapy, biological therapy, immunotherapy, and surgical resection [8,9]. Oxaliplatin (OXA) is one of the most widely used clinical drugs, which can bind to DNA to hinder its replication to induce tumor cell death [10]. OXA has the advantages of causing mild depression of bone marrow, low-grade gastrointestinal adverse reaction, and remarkable therapeutic effect. But at the same time, it also has the disadvantages of strong peripheral neurotoxicity and immune syndrome suppression, which affects patients’ quality of life and the drug’s clinical applicability [11,12,13].

Cancer stem cells (CSCs), also known as tumor stem cells, are an important subgroup of cancer histiocytes that have the special properties of self-renewal, clonal initiation, and resistance to death [14,15]. They can participate in tumor development through long-term clone propagation, distant metastasis, angiogenesis, and evasion from the immune system, with changes in the expression of many biomarkers, such as CD44, CD117, CD133, SOX2, OCT4, NANOG, and ALDH1 [16,17,18,19]. Epithelial mesenchymal transition (EMT), one of the main factors causing tumor cell metastasis, refers to the process in which epithelial cells lose their cell–cell adhesion properties and acquire stromal cell characteristics, transforming into invasive mesenchymal cells and actively participating in many biological processes such as tumor cell occurrence, invasion, and drug resistance [20,21,22]. Above all, EMT is also one of the characteristic tumor stemness traits [23]. It has been reported that ZO1, E-cadherin, and Snail are representative markers of EMT [20,24]. Due to the resistance to conventional anti-cancer therapy, CSCs can recover to proliferate and result in unideal treatment outcomes [25,26]. Therefore, finding effective therapeutic strategies to eliminate tumorigenic CSC subpopulations holds great promise to improve the activity of anti-cancer drugs, providing new hope for conquering cancers [27,28].

S100A8, also known as MRP8, belongs to the S100 family of Ca^2+^-binding proteins and is usually presented as heterodimers with homologous S100A9 proteins [29]. Studies have confirmed that S100A8 is upregulated in various tumor cells, including colorectal cancer, anaplastic thyroid carcinoma, breast cancer, lung cancer, and acute myeloid leukemia, and is involved in regulating cell proliferation, metastasis, invasion, drug resistance, angiogenesis, and immunosuppression [30,31,32,33,34,35,36,37]. However, there are currently limited reports on the correlation between S100A8 and CSCs or their stemness characteristics [38,39].

Natural compounds refer to chemical substances with significant pharmacological activity that exist widely in plants, fungi, and marine animals. These compounds have been developed into immunosuppressants, anti-cancer agents, nutraceuticals, and oxidation inhibitors [40,41,42,43]. For instance, irinotecan, paclitaxel, and bleomycin are typical examples of anti-cancer drugs derived from natural products [44,45]. Compared with plants or animals, fungal metabolites have richer variety, stronger controllability, and greater specificity, which can be used for low-cost, high-efficiency, and large-scale industrial production, thus having good development prospects [46,47]. In our previous research, we obtained an epitetrathiodioxopiperazine compound called secoemestrin C (Sec C) from *Emericella* sp. via microbial fermentation, which was isolated for the first time in 1997 [48]. However, only a few articles have reported its effects on immunosuppression, the downregulation of isocitrate lyase activity, and the induction of endoplasmic reticulum stress [48,49,50,51]. Our early studies demonstrated that Sec C had a significant killing effect on several tumor cell lines with low toxicity in vivo; thus, we wanted to explore the concrete regulatory pathways of this compound on colorectal tumor cells in more depth [51]. Finally, we confirmed that Sec C had a strong inhibitory effect on the stemness features of colorectal tumor cells. Meanwhile, S100A8, as well as the p38 signal pathway, played an inevitable part in the maintenance of CSCs and the Sec C killing process. This provided new insights and proof that Sec C could serve as a promising anti-cancer prodrug and S100A8 could be a new drug target as well as a biomarker for cancer treatment and diagnosis.

## 2. Material and Methods

### 2.1. Reagents

The Cell-Light 5-ethynyl-2′-deoxyuridine (EdU) Apollo 488 in vitro kit (C10310-3) was purchased from RiboBio (Guangzhou, China). Oxaliplatin (OXA) was purchased from the Cancer Hospital of the Chinese Academy of Medical Sciences (Beijing, China). MTT was purchased from Sigma-Aldrich (St. Louis, MO, USA). Anti-GAPDH, anti-NANOG, anti-Snai1, anti-p38, and anti-p-p38 antibodies were purchased from Cell Signaling Technology (Danvers, MA, USA). Anti-OCT4, anti-SOX2, anti-ZO1, anti-E-cadherin, anti-CD133, and anti-Ki67 antibodies were purchased from Proteintech Group, Inc. (Rosemont, IL, USA). Anti-S100A8 antibodies were purchased from Beyotime (Nantong, China).

### 2.2. Cell Culture

The human colorectal tumor cell lines HCT8, HT29, HCT116, HCT15, RKO, and CW2 were purchased from the Cell Culture Center of Peking Union Medical College (PUMC, Beijing, China). The human colorectal tumor cell lines HCT8/L with OXA resistance were purchased from ShangHai MEIXUAN Biological science and technology LTD (Shanghai, China). The human normal colon epithelial cell line NCM460 was purchased from Beijing Mintai Yuan Technology Co., Ltd. (Beijing, China). HCT8, HCT15, CW2, HCT8/L, and NCM460 cells were cultured in RPMI 1640 (MacGene, Beijing, China). HT29 was cultured in DMEM/F12 (1:1) (Gibco, CA, USA). HCT116 was cultured in DMEM (MacGene). RKO was cultured in MEM (Gibco). All cells were cultured in medium supplemented with 10% fetal bovine serum (Gibco, CA, USA), 100 mg/mL streptomycin, and 100 U/mL penicillin (MacGene) at 37 °C in 5% CO_2_. All cell lines were maintained and used in ≤20 passages.

### 2.3. Cell Transfection

S100A8 and p38 plasmids were purchased from Sino Biological (Beijing, China), siRNA of S100A8 was purchased from RiboBio (Guangzhou, China), and Sh S100A8 plasmid was designed by and purchased from Sangon Biotech (Shanghai, China).

The stable cell lines were selected using hygromycin and neomycin. In brief, the S100A8 overexpression plasmid or Sh S100A8 plasmid was transfected separately into the target cells. After 48 h of the transfection, cells were cultured in the selective medium containing hygromycin or neomycin. Through the infinite dilution method, stable single clones were selected and expanded.

HCT8 and HT29 colorectal tumor cells were seeded in the plates and transfected with plasmids or siRNA at 70–80% confluence using Lipofectamine 2000 or Lipofectamine RNAiMAX transfection reagent (Invitrogen, Waltham, MA, USA).

### 2.4. Cell Viability Assay

Wild-type and stably transfected cells were seeded in 96-well plates at a density of 8000 cells per well, and treated with Sec C or OXA in gradient concentrations. After incubating at 37 °C with 5% CO_2_ for 48 h, cell growth was assessed by MTT assay. Namely, the MTT solution was incubated with cells for 3.5 h at 37 °C with 5% CO_2_, and after the purple precipitate was dissolved by DMSO, the optical density at 490 nm was measured using a spectrophotometer. The cell survival rate was calculated at each concentration, and the IC_50_ values were calculated by SigmaPlot. Each assay was replicated three times.

### 2.5. Colony Formation Assay

Cells were seeded in 6-well plates at a density of 1000 cells per well and treated with Sec C or OXA with different concentrations. For wild-type cells, they were incubated with drugs for 2 weeks while stably transfected cells were treated with drugs for 6 h (S100A8 downregulated cells) or 24 h (S100A8 overexpressed cells), and then transferred into the fresh medium to proliferate for 2 weeks. After being washed softly with PBS buffer, the cells were fixed in methanol for 10 min at ambient temperature and then stained with 0.1% crystal violet (Beyotime, Nantong, China) for 30 min. After rinsing with PBS buffer and air drying, colonies containing more than 50 cells were counted and scanned. Each assay was replicated three times.

### 2.6. Soft Agar Assay

Cells were seeded in 6-well plates at a density of 350,000 cells per well and treated by Sec C for 3 h (stable S100A8 downregulated cells) or 24 h (wild-type cells and stable S100A8 overexpressed cells), and then the cells were collected and counted before being transferred into new 6-well plates loaded with 1.2% agarose medium, on which the cells were mixed with 0.7% agarose medium and cultured at a density of 5000 cells per well. After 2 weeks, colonies were stained with 0.1% crystal violet (Beyotime, Nantong, China) for 30 min, and after being washed with PBS buffer, the viable colonies were analyzed under the microscope. Each assay was replicated three times.

### 2.7. Sulforhodamine B (SRB) Stain Assay

After being washed softly with PBS buffer, the Sec C- or OXA-treated cells were fixed by 10% TCA solution at 4 °C, and then stained with SRB dye for 1 h at ambient temperature. Then, 1% acetic acid was used to clear redundant dye, and after drying by air, Tris-base (pH 10.5) solution dissolved the dye combined with cells, and the optical density at 540 nm was measured using a spectrophotometer. Each assay was replicated three times.

### 2.8. Wound Healing Assay

Cells were seeded in 6-well plates and cultured to reach 80% confluence. Then, a 1 mL pipette tip was used to scratch the plates to create a wound in the middle of the plate. Sec C of different concentrations was added to co-culture with cells with 3% FBS medium. Images at 0 and 48 h were captured using a microscope, and the cells’ migration rate was calculated by the formula (Migration area/initial area) × 100%. Each assay was replicated three times.

### 2.9. Sphere Formation Assay

Cells were plated into 6-well ultralow attachment plates (Corning) with sphere medium at a density of 20,000 viable cells per well. Sec C of different concentrations was added into the sphere medium to incubate with cells for 24 h. The spheres with ≥60 μm diameter were counted and CCK8 solution (Beyotime, Nantong, China) was added into the medium to incubate for 1 h at 37 °C with 5% CO_2_ to quantify the cells. The optical density at 450 nm was measured using a spectrophotometer. The sphere medium contained EGF (20 ng/mL, MCE), LIF (20 ng/mL, Cell Signaling Technology), bFGF (10 ng/mL, Cell Signaling Technology), insulin (5 μg/mL, MCE), streptomycin/penicillin (1%, MacGene), BSA (0.1%, MCE), and B27 (1×, Gibco). Each assay was replicated three times.

### 2.10. 5-Ethynyl-2′-Deoxyuridine (EdU) Assay

The EdU assay was performed to assess active DNA synthesis using an EdU Apollo 488 Kit (RiboBio, China). Wild-type or stably transfected cells were seeded in 24-well coverslips at 40,000 cells per well and Sec C was added to treat the cells for 24 h. After incubation with EdU solution, the cells were fixed by 4% paraformaldehyde (Beyotime, China) and stained with Apollo 488 according to the manufacturer’s protocol (RiboBio, China). DAPI (Vector Labs, CA, USA) was used to show the nucleus. Images were acquired using a Leica SP2 confocal microscope (Leica Microsystems, Exton, PA, USA) and the ratio of EdU-positive cells was analyzed by ImageJ (Version 1.53).

### 2.11. Western Blotting

Cells were collected and lysed in RIPA buffer (Beyotime, China) containing PMSF (Beyotime, China), and the protein concentrations were quantified using a BCA Protein Assay Kit (Beyotime, China). Protein lysates were separated by 10% or 12% SDS-PAGE gels and transferred onto PVDF membranes (Millipore, Danvers, MA, USA), which were then blocked by 5% skimmed milk for 2 h at ambient temperature. The membranes were first cultured with the indicated antibodies overnight at 4 °C, including GAPDH (1:1000), ZO1 (1:1000), E-cadherin (1:1000), Snail (1:800), OCT4 (1:800), SOX2 (1:800), NANOG (1:800), S100A8 (1:1000), p38 (1:1000), and p-p38 (1:1000). After washing with TBS-T, the respective IgG-HRP-labeled secondary antibodies (1:5000, Zhongshan Goldenbridge, Beijing, China) were used to incubate the membranes for 2 h at ambient temperature. The FluorChem HD2 imaging system (Protein Simple, CA, USA) was applied to visualize the target protein bands.

### 2.12. RNA Sequencing (RNA-Seq)

The RNA of Sec C-treated cells and S100A8-overexpressed cells was extracted in accordance with the procedures of the RNA extraction kit (Invitrogen). Quality control of the raw reads enabled the downstream analysis to be based on the clean reads with high quality. The sequencing process was performed on a BGISEQ-500RS platform. The raw sequencing data are all available in the Sequence Read Archive database linked with BioProject F20FTSNCKF2720_POEubyE and F22FTSNCKF1231_POEvzxkT.

### 2.13. Flow Cytometry

HCT8 cells were treated with 1 and 2 μM Sec C for 24 h, then washed three times with PBS buffer and collected for 5% BSA incubation at 4 °C. After washing softly with PBS buffer again, the cells were co-incubated with anti-CD133 antibody (1 μg/1 mL) for 40 min in the dark at room temperature. A BD FACSCalibur flow cytometer (BD Bio sciences, San Jose, CA, USA) was used to measure the colorectal tumor stem cells.

### 2.14. Immunofluorescence Assay

HCT8 cells were seeded in 24-well coverslips, fixed with 4% paraformaldehyde and permeabilized by 0.5% Triton X-100, and then incubated with 10% goat serum (Beyotime, China). Next, the cells were incubated overnight at 4 °C with antibodies against S100A8 (1:100) and p38 (1:100). After being washed with PBS buffer, the Alexa Fluor 488- or 647-labeled secondary antibodies (1:500) were incubated with the cells for 1 h in the dark. The slides were stained with DAPI (Vector Labs, CA, USA) to visualize the nuclei. Images were acquired using a Leica SP2 confocal microscope (Leica Microsystems, Exton, PA, USA) and analyzed with ImageJ (Version 1.53).

### 2.15. Immunohistochemistry (IHC) Analysis

Tumor slides were first incubated with 1 mM Tris-EDTA (pH 8.0) and 3% hydrogen peroxide, respectively, for antigen repair and blockage of endogenous peroxidases. Then, the tissues were blocked with 10% goat serum for 30 min at ambient temperature. The S100A8, p38, and Ki67 antibodies were separately added for incubation overnight at 4 °C, and after being washed with PBS buffer, the slides were incubated with a biotin-labeled goat anti-rabbit IgG (Beyotime, China) for 40 min at ambient temperature. DAB staining solution and hematoxylin were used to observe the target protein staining. Images were obtained using a Digital Pathology System (3D HISTECH, Budapest, Hungary) and IHC score analysis was undertaken by ImageJ (Version 1.53).

### 2.16. Xenograft Tumor Model

Six-week-old BALB/c nude mice were purchased from SPF Biotechnology Co., Ltd. (Beijing, China) and randomly separated into indicated groups (*n* = 5 per group). HCT8 cells were resuspended in the serum-free medium and subcutaneously injected (10^7^ cells/tumor) into the left back of each mouse. Then, the mice were weighed (g), and the tumor width (W) and length (L) were measured by vernier calipers every 2 days. Tumor volume was calculated based on the formula V = 0.5 × L × W^2^. When the tumor volume reached 90 mm^3^, Sec C (2.5 and 5 mg/Kg) or OXA (5 mg/Kg) was given by intraperitoneal injection. When the largest tumor grew over 2000 mm^3^, the mice were sacrificed and the tumors were excised and weighed. The main organs were used for HE staining and the tumors were used for IHC staining. The care and treatment of the experimental animals were conducted on the basis of the institutional guidelines at the Experimental Animal Center of the Chinese Academy of Medical Sciences.

### 2.17. Statistical Analyses

All values in the figures and the text were derived from at least 3 independent experiments and expressed as means ± standard deviation. The statistical analyses were performed by GraphPad Prism (Version 6.01). Student’s t-test and two-way analysis of variance were applied to analyze statistical differences between independent groups. Statistical significance was set at * *p* < 0.05, ** *p* < 0.01, *** *p* < 0.001, and **** *p* <0.0001. NS indicated that no significance was noted.

## 3. Results

### 3.1. Sec C Effectively Inhibits the Proliferation of Colorectal Tumor Cells

**Figure 1:**
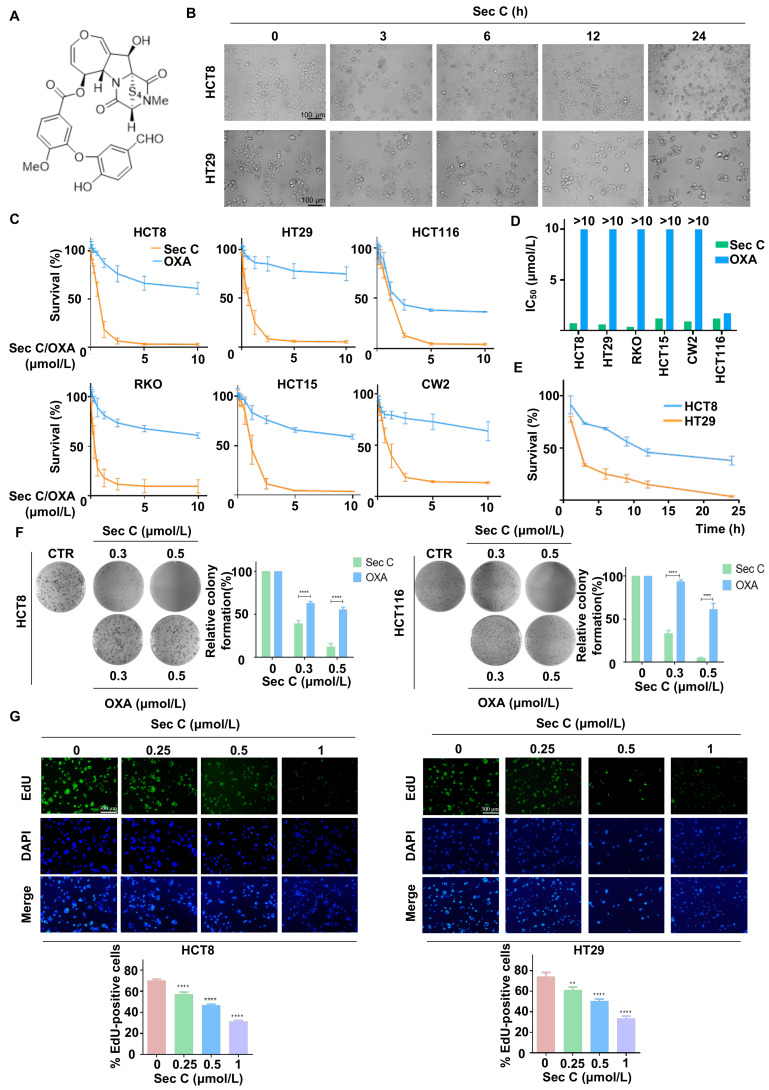
Sec C exhibits strong anti-colorectal tumor activity dose-dependently. (**A**) The chemical structure of Sec C. (**B**) The cell morphology of HCT8 and HT29 cells was observed after the treatment with 2.5 μmol/L Sec C for the indicated time points. Scale bar, 100 μm. (**C**) Different colorectal tumor cells were treated with the specific concentrations of Sec C or OXA for 48 h, and cell survival was detected by MTT assay. The dose-dependent curves were drawn via GraphPad Prism (Version 6.01). (**D**) IC_50_ of Sec C and OXA in different colorectal tumor cell lines. (**E**) The vitality of HCT8 and HT29 cells was determined via MTT assay after treatment with 2.5 μmol/L Sec C for the indicated time periods. A time-dependent curve was plotted by GraphPad Prism (Version 6.01). (**F**) HCT8 and HCT116 cells were seeded in 6-well plates and Sec C was added for incubation. Then, visible colonies containing more than 50 cells were counted. (**G**) HCT8 and HT29 cells were treated with Sec C for 24 h, and cell proliferation was detected with an EdU assay. Scale bar, 300 μm. The data are shown as mean ± SD (*n* = 3). ** *p* < 0.01, and **** *p* < 0.0001.

Sec C is an epitetrathiodioxopiperazine compound extracted from endophytic fungi, with a relative molecular weight of 662 g/mol (Figure 1A). We observed that Sec C showed strong anti-cancer effects in HCT8 and HT29 colorectal tumor cells. Morphological images indicated that both HCT8 and HT29 cells became round within 3 h of the co-incubation of 2.5 μmol/L Sec C, and 80% of HT29 cells became nonadherent within 12 h, suggesting that the colorectal tumor cells were nonviable (Figure 1B). To evaluate the dose-dependent effect of Sec C, different concentrations of 0, 0.075, 0.1500, 0.3125, 0.625, 1.25, 2.5, 5.0, and 10.0 μmol/L Sec C were separately added to six types of colorectal tumor cells (HCT8, HT29, HCT116, RKO, HCT15, and CW2 cells) for 48 h. The clinical drug OXA acted as the control. The results finally showed that Sec C significantly inhibited cell growth dose-dependently, and we obtained the IC_50_ from survival rate–concentration curves. For example, regarding HCT8, the cell survival rate was approximately 55% after the treatment of 0.625 μmol/L Sec C for 48 h, which dropped to 18% when the concentration rose to 1.25 μmol/L (Figure 1C). Upon comparing the IC_50_ between Sec C and OXA, we found that, with the exception of HCT116, the remaining five types of tumor cells all achieved a considerably higher IC_50_ of OXA (>10 μmol/L). In contrast, Sec C had a prominent inhibitory effect with less than 1.5 μmol/L in terms of the IC_50_. In other words, there was an almost six-fold difference between OXA and Sec C, even for RKO cells, and the killing activity of Sec C was more than 30 times stronger than that of OXA (Figure 1D and Figure S1A). We also detected the Sec C activity on human normal colon epithelial cells NCM460; the results showed that the IC_50_ of Sec C on them was 4.0859 μmol/L, namely, several times higher than that on tumor cells (Figure S1B). Meantime, the time-dependent effect of Sec C was investigated at 1, 3, 6, 9, 12, and 24 h (Figure 1E). As for HCT8, approximately 73% of tumor cells were viable after 3 h; in addition, 55% and 37% of cells were viable after 9 and 24 h, respectively.

Given that the colony formation assay is a significant method for the analysis of cytotoxicity, we utilized it to further assess the inhibitory effects of Sec C. The results indicated that Sec C repressed the colony formation of tumor cells dose-dependently, and this was better than that achieved by OXA. Taking HCT8 cells as an example, at 0.3 μmol/L, the colony formation rate of treated cells was approximately 39% of the control under Sec C treatment, and was 63% under OXA treatment, which was even accompanied by a bigger cloning size. The colony formation rate of 0.5 μmol/L Sec C-treated cells was approximately 12% of the control, while 0.5 μmol/L OXA-treated cells were approximately 55% of the control (Figure 1F and Figure S1C). EdU is a type of thymidine analog that incorporates into DNA molecules during active DNA synthesis and tumor cell proliferation. Apollo fluorescent dyes can conjugate with EdU, so the DNA replication and proliferation level of tumor cells can be explored by detecting the intensity of Apollo fluorescent dyes [52]. Next, we used an EdU assay to determine the activity of Sec C on DNA replication and discovered that the EdU-positive cell rate decreased along with increasing Sec C concentrations (Figure 1G and Figure S1D), indicating a potent effect of Sec C on DNA replication.

All of these findings confirmed that Sec C had distinct inhibitory effects on colorectal tumor cells and induced obvious tumor cell death, suggesting that Sec C could be developed as a prodrug for further clinical treatment for cancers.

### 3.2. Sec C Kills Highly Drug-Resistant Colorectal Tumor Cells

**Figure 2:**
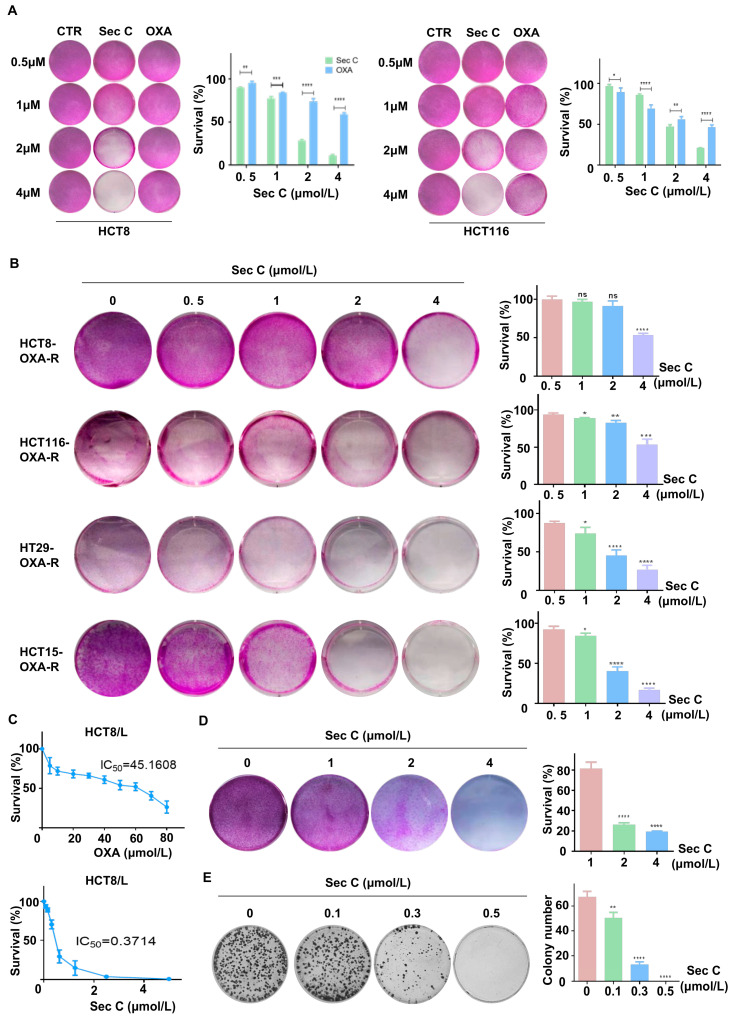
Sec C is effective at killing OXA-resistant cells. (**A**) HCT8 and HCT116 cells were treated with the Sec C or OXA for 48 h, and cell viability was detected with SRB assay. (**B**) HCT8, HCT116, HT29, and HCT15 cells were constantly stressed by OXA, and then the viability of OXA-R cells after being treated with Sec C was detected with SRB assay. (**C**) HCT8/L cells were treated with the indicated concentrations of OXA or Sec C for 48 h, and cell viability was detected by MTT assay. The dose-dependent curves were drawn using GraphPad Prism (Version 6.01). (**D**) HCT8/L cells were treated with Sec C for 48 h, and cell viability was detected with SRB assay. (**E**) HCT8/L cells were seeded in 6-well plates and Sec C was added for co-incubation. Then, visible colonies containing more than 50 cells were counted. The data are shown as mean ± SD (*n* = 3). * *p* < 0.05, ** *p* < 0.01, *** *p* < 0.001, and **** *p* < 0.0001. NS indicated that no significance was noted.

Previous results found that Sec C had a clear killing action on colorectal tumor cells, and showed a stronger inhibitory activity at high concentrations. Next, we enhanced the density of tumor cells in six-well plates and co-incubated them with increasing concentrations of Sec C or OXA (0, 0.5, 1, 2, and 4 μmol/L) for 48 h. The results revealed that the high concentrations of Sec C killed most of the cells in the wells of view. Concerning HCT8, 4 μmol/L Sec C repressed cell proliferation to approximately 10% of the control, while 4 μmol/L OXA resulted in a survival rate of 58% of the control. This showed the powerful suppressive effect of Sec C (Figure 2A and Figure S2A).

Drug resistance is one of the main reasons why tumors are difficult to completely eliminate; thus, they can even relapse and grow again, which is also one of the tumor stemness traits [53]. We treated wild-type colorectal tumor cells with OXA for 48 h to collect specific cells with enhanced resistance to OXA. We added increasing concentrations of Sec C (0, 0.5, 1, 2, and 4 μmol/L) for 48 h to test the cell viability. We observed that Sec C also killed these higher OXA-resistant cells dose-dependently. For HCT8, 4 μmol/L Sec C suppressed the cell growth down to 52% of the control cells. Other cell lines also showed the same status, which meant that Sec C might have an inhibitory effect on drug-resistant tumor cells (Figure 2B). HCT8/L cells are a type of specially cultivated cells with definite OXA resistance. We separately treated HCT8/L cells with Sec C and OXA using different doses to detect the IC_50_, and we found that the IC_50_ of Sec C was 0.3714 μmol/L while the IC_50_ of OXA was 45.16 μmol/L (Figure 2C). We enhanced the density of HCT8/L cells in six-well plates and co-incubated them with an increasing dose of Sec C. As expected, 4 μmol/L Sec C also effectively killed HCT8/L cells with a 19% survival rate of the control cells (Figure 2D). The colony formation assay also indicated that Sec C had a significant inhibitory effect on these drug-resistant cells, and even 0.5 μmol/L Sec C completely restrained HCT8/L cell proliferation in the wells of view (Figure 2E). All of these results indicated that Sec C had a clear killing action on drug-resistant tumor cells.

### 3.3. Sec C Restrains the Stemness Features of Colorectal CSCs

Research has reported that CSCs are the main factors driving tumor initiation and progression, which are closely related to the proliferation, invasion, drug resistance, and self-renewing capacity of tumor cells [54,55]. Based on the fact that Sec C had a strong inhibitory effect on colorectal tumor cells, even drug-resistant cells, we next wanted to test whether Sec C could suppress CSCs and their stemness signatures.

**Figure 3:**
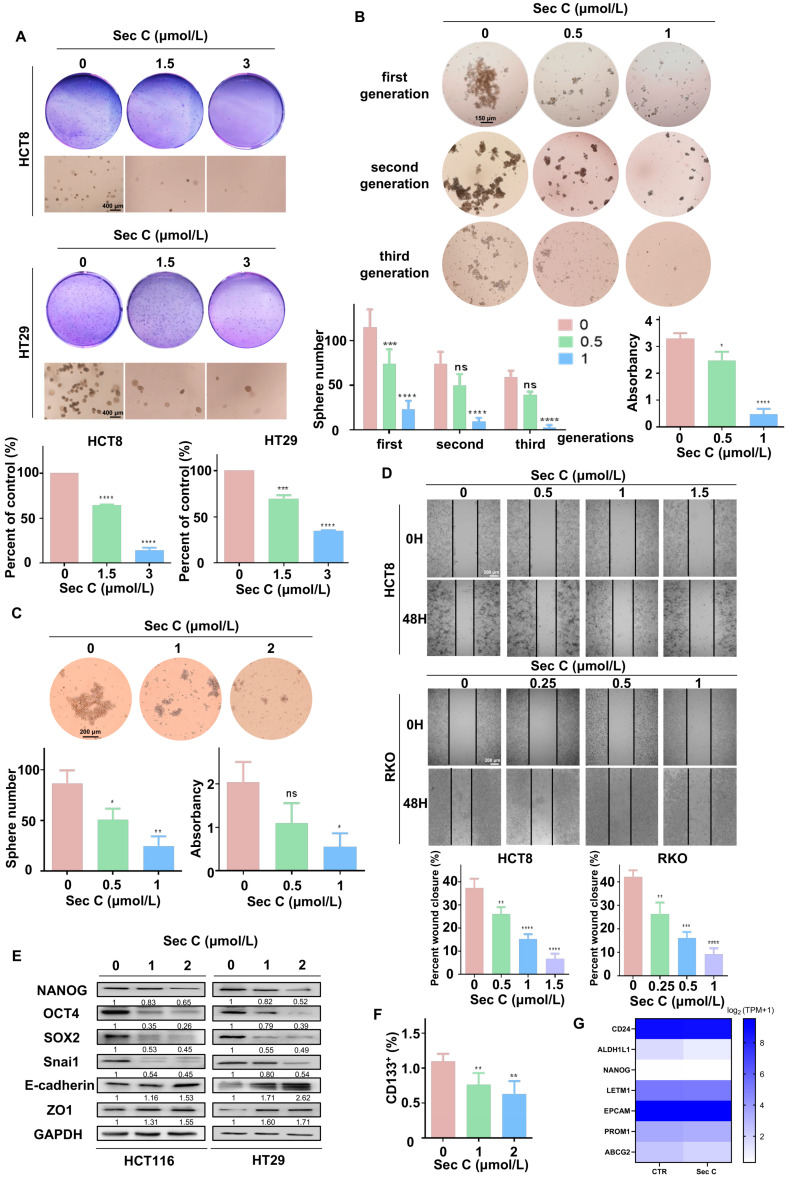
Sec C significantly inhibits colorectal CSCs and their stemness traits. (**A**) The visible colonies of HCT8 and HT29 cells cultivated in soft agar. Circular images, naked-eye observation. Square images, microscopic observation. Scale bar, 400 μm. (**B**) The grown spheres (diameter ≥ 60 μm) of HT29 cells were observed under a microscope. Scale bar, 150 μm. (**C**) The HT29 sphere cells (diameter ≥ 60 μm) were counted after being treated with Sec C under a microscope. Scale bar, 200 μm. (**D**) Representative images of the wound from HCT8 and RKO cells were recorded by microscope. Scale bar, 200 μm. (**E**) Western blotting for detecting the stemness and EMT markers in HCT116 and HT29 cells. (**F**) The changes in the proportion of CSCs from HCT8 cells were measured by FCM. (**G**) The stemness proteins decreased due to Sec C treatment according to RNA-seq analysis. The data are shown as mean ± SD (*n* = 3). * *p* < 0.05, ** *p* < 0.01, *** *p* < 0.001, and **** *p* < 0.0001. NS indicated that no significance was noted.

Some malignant tumor cells can not only grow in an adherent state, but also proliferate under suspension conditions. The ability of tumor cells to form clones in soft agar can reflect their potential for malignant hyperplasia. To examine this, we carried out a soft agar assay. It was apparent that Sec C dramatically inhibited the stereoscopic growth of HCT8 and HT29 cells. For instance, for HCT8 cells, 1.5 μmol/L Sec C led to a 64% cell growth rate of the control cells, and 3 μmol/L Sec C only left 13% of cells to grow (Figure 3A).

The sphere formation assay is a gold standard to reflect the stemness traits of CSCs. Just like the soft agar assay, we added dose-dependent Sec C to treat HT29 cells for 24 h and transferred them into six-well low-attachment plates to culture. As the spheres grew, we collected and passaged them to the second generation, which was followed by the third generation in the same way. We found that 1 μmol/L Sec C obviously restrained the self-renewing ability of tumor cells, which were unable to grow into spheres in the third generation. Only around 4% of cells compared with the control group held sphericity, indicating the strong suppression of Sec C on the self-renewal ability of the tumor cells (Figure 3B). At the same time, we cultured HT29 cells in six-well low-attachment plates until the spheres grew; then, we added various concentrations of Sec C to co-incubate with the spheres for 24 h. In the end, the number of spheres as well as CCK8 quantification showed that Sec C also restrained the maintenance of spheres with more stemness traits dose-dependently. In detail, compared with the control group, 0.5 μmol/L Sec C left 58% of the spheres to survive, and 1 μmol/L Sec C led to 28% of the spheres surviving with a smaller size and a loose shape (Figure 3C).

Migration ability is an important part of the stemness traits of tumor cells. As shown, under 1.5 and 1 μmol/L Sec C, it was difficult for the blank area to be covered by cells, and only 17% and 20% of the migration rates of the control group were calculated for HCT8 and RKO cells, respectively, which demonstrated the reliable repression of Sec C on the cell metastasis ability (Figure 3D). Moreover, Western blotting showed that 2 μmol/L Sec C effectively changed the expression of typical stemness traits makers. In other words, Sec C had the strength to negatively regulate the stemness features and EMT process of the tumor cells (Figure 3E).

In CSCs, some extracellular or intracellular biomarkers are used for distinguishment and identification [15,56]. We treated HCT8 cells with Sec C for 24 h, and then tested the changes in CSCs through CD133. As expected, Sec C was useful for decreasing the population of CSCs dose-dependently (Figure 3F). What is more, we extracted RNA from Sec C-treated HT29 sphere cells and carried out RNA-seq analysis, and we found that some iconic markers representing stemness properties were significantly reduced (Figure 3G).

From these findings, we confirmed that Sec C had a clear inhibitory effect on colorectal CSCs and their stemness features, including growth, drug resistance, self-renewal, and migration. We next wanted to deeply explore the specific regulatory mechanisms in order to fully elucidate the functions and roles of Sec C in colorectal tumor cells.

### 3.4. S100A8 Confronts the Suppressive Effect of Sec C on Colorectal Tumor Cells

**Figure 4:**
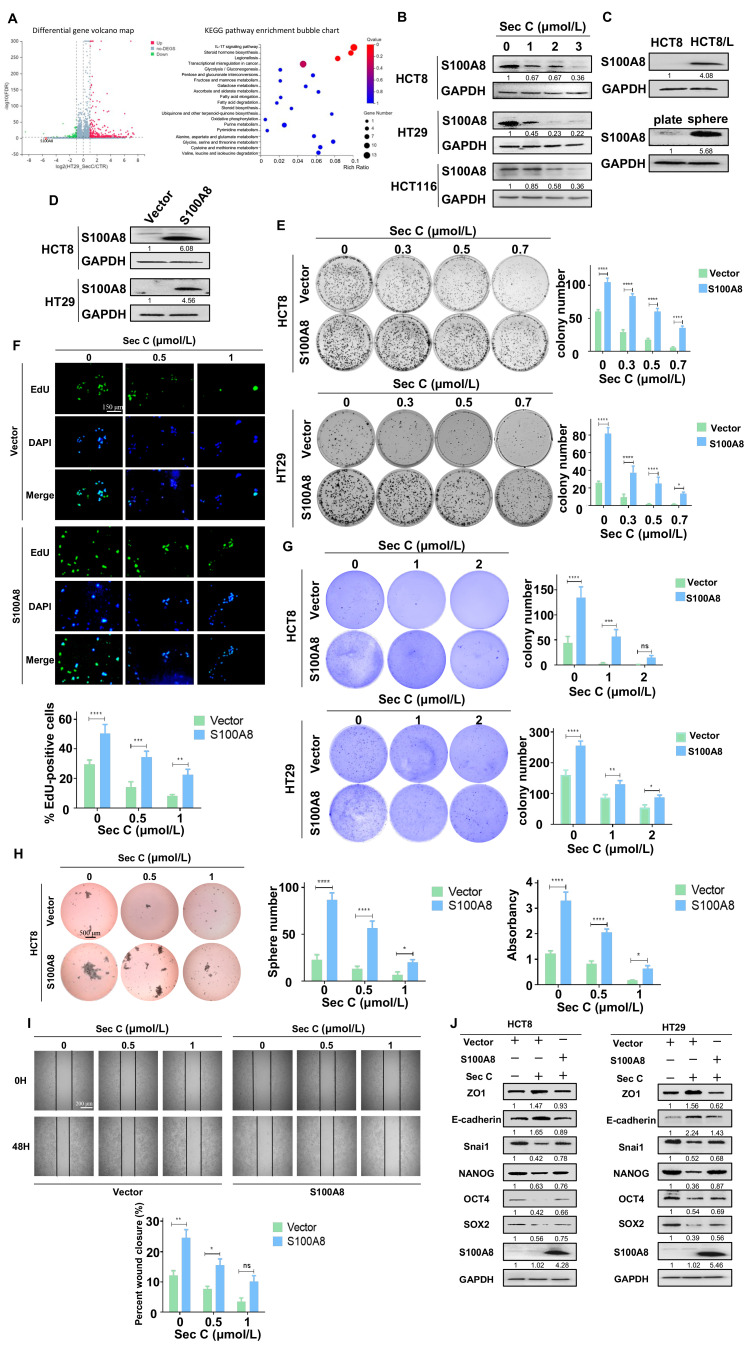
S100A8 reverses the effect of Sec C on colorectal tumor cells. (**A**) The genes had changed and the activated transduction pathways of Sec C-treated HT29 sphere cells. (**B**) HCT8, HT29, and HCT116 were treated by Sec C to detect the changes in S100A8 by Western blotting. (**C**) The expression of S100A8 in HCT8/L and sphere cells. (**D**) The expression of S100A8 in HCT8 and HT29 cells after being stably transfected by S100A8 plasmid. (**E**) The stable S100A8-overexpressing HCT8 and HT29 cells were seeded in 6-well plates and treated with Sec C. The visible colonies were counted as shown. (**F**) The S100A8-overexpressing HCT8 cells were treated with Sec C, and the DNA replication level was examined by EdU assay. Scale bar, 150 μm. (**G**) The visible colonies of the stable HCT8 and HT29 cells in soft agar were counted under a microscope. (**H**) The stable HCT8 cells were treated with Sec C and cultured with serum-free medium, and then the spheres (diameter ≥ 60 μm) were counted. Scale bar, 500 μm. (**I**) Representative images of the wound from S100A8-overexpressing HCT8 cells were recorded by microscope. Scale bar, 200 μm. (**J**) The Western blotting of S100A8-overexpressing HCT8 and HT29 cells for stemness and EMT markers. Vector, the plasmid empty vector. S100A8, the overexpressed plasmid group. The data are shown as mean ± SD (*n* = 3). * *p* < 0.05, ** *p* < 0.01, *** *p* < 0.001, and **** *p* < 0.0001. NS indicated that no significance was noted.

To further clarify the mechanism of Sec C, we compared the difference between Sec C-treated HT29 sphere cells and control cells via RNA-seq analysis. Among the many genes that changed expression due to Sec C, we found that S100A8 declined most obviously (Figure 4A). We detected S100A8 in Sec C-treated cells and found that 1–3 μmol/L Sec C significantly inhibited the expression of S100A8 protein dose-dependently (Figure 4B). We collected OXA-resistant HCT8/L cells and HT29 sphere cells, which both presented more stemness traits, to compare them with normal tumor cells. We found that S100A8 was highly expressed in those specific cells, implying that S100A8 might have a close relationship with stemness features in colorectal tumor cells (Figure 4C). Next, we wanted to explore whether S100A8 played an important role in the Sec C killing process of colorectal tumor cells. From previous results, we know that HCT8 cells have little S100A8 expression, while HT29 cells have more S100A8 expression. Consequently, we constructed stable S100A8-overexpressing HCT8 and HT29 cell lines (Figure 4D). First, we utilized a colony formation assay to observe the proliferation ability of the tumor cells. Both HCT8 and HT29 cells showed that upregulated S100A8 enhanced the resistance of cells to Sec C, such as HCT8; in detail, overexpressed S100A8 contributed to elevated colony potential by 289%, 344%, and 645% at 0.3 μmol/L, 0.5 μmol/L, and 0.7 μmol/L Sec C of the control group, respectively, and the same reverse function was confirmed by HT29 cells (Figure 4E). We also carried out an EdU assay to test the DNA replication level of tumor cells, and we found that S100A8 enhanced DNA synthesis to counter Sec C treatment. Overexpressed S100A8 increased EdU-positive cells by 242% and 276% at 0.5 and 1 μmol/L Sec C on HCT8 cells, respectively. Meanwhile, the same phenomenon was also found with HT29 cells (Figure 4F and Figure S3A). The stereoscopic proliferation ability of tumor cells was also tested by soft agar assay. The results showed that the number of colonies greatly increased after S100A8 overexpression, and the shape was larger than that of the control group. For HCT8 cells, the number of colonies rose by 212% at 2 μmol/L Sec C, and the same reversed effect was also found for HT29 cells (Figure 4G). To assess sphere formation ability, we left the Sec C-treated cells for 24 h and put them in a six-well low-attachment plate for cultivation. It was obvious that the spheres grew much larger after S100A8 upregulation and the number greatly improved by 150% under treatment with 1 μmol/L Sec C on HCT8 cells. Meanwhile, the HT29 cell groups also showed larger spheres, even with the pressure of Sec C. This fully proved that S100A8 enhanced the self-renewal ability of tumor cells to oppose Sec C (Figure 4H and Figure S3B). We also examined the migration of tumor cells with highly expressed S100A8. It was evident that an approximately 34% increment was observed under 1 μmol/L Sec C after 48 h on HCT8 cells. Together with HT29 cells, we believed that S100A8 promoted the movement of tumor cells and played an obstructive role in the inhibitory effect of Sec C on tumor cell migration (Figure 4I and Figure S3C). Meanwhile, Western blotting showed us that the markers of EMT and stemness traits were all changed by upregulating S100A8 in HCT8 cells and HT29 cells, which meant that S100A8 overexpression reversed Sec C to promote the stemness features and EMT process of the tumor cells (Figure 4J).

### 3.5. Downregulated S100A8 Increases the Sensitivity of Colorectal Tumor Cells to Sec C

**Figure 5:**
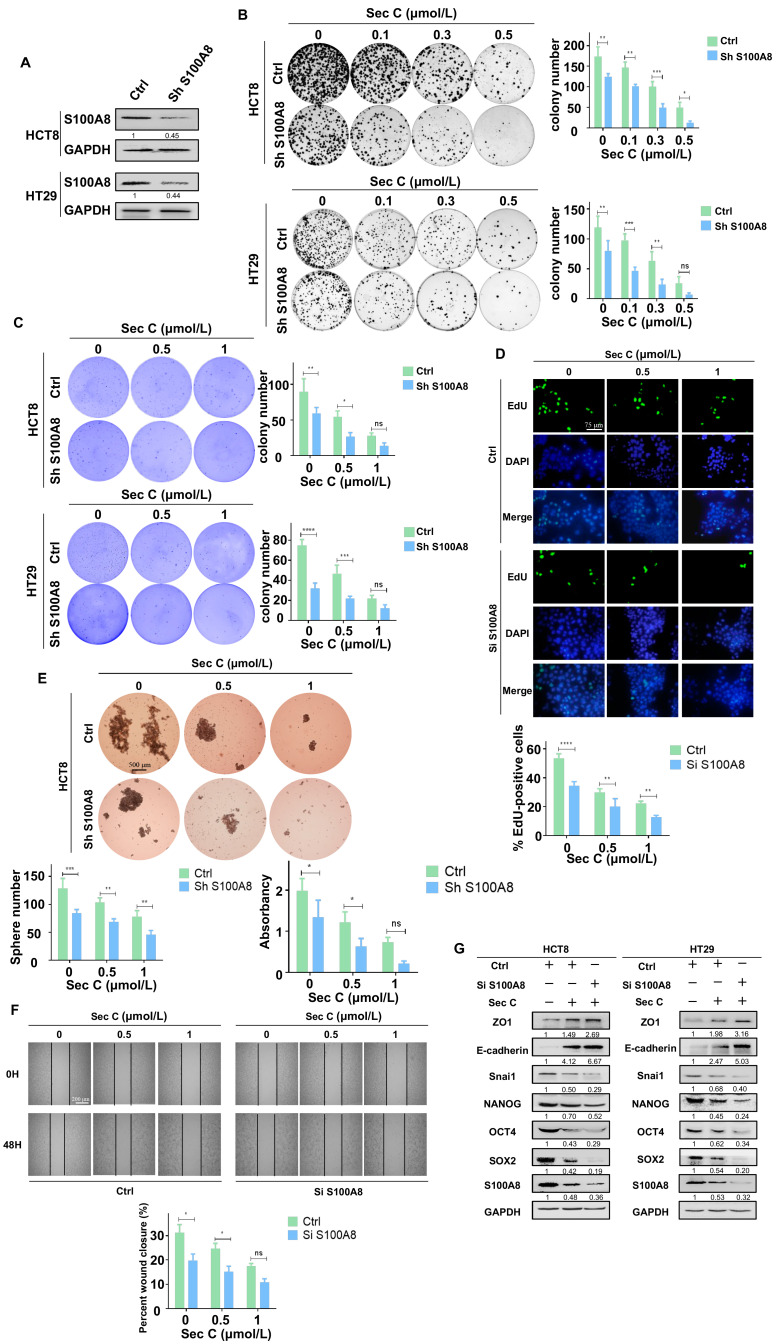
Downregulated S100A8 increases the sensitivity of colorectal tumor cells to Sec C. (**A**) The expression of S100A8 in HCT8 and HT29 cells after being stably transfected with Sh S100A8 plasmid. (**B**) The visible colonies of the stable S100A8-knockdown HCT8 and HT29 cells were counted as shown. (**C**) The stable HCT8 and HT29 cells were treated with Sec C and then seeded in soft agar. The visible colonies were counted after being stained. (**D**) The S100A8-knockdown HCT8 cells were treated with Sec C, and cell proliferation was detected with EdU assay. Scale bar, 75 μm. (**E**) The stable S100A8-knockdown HCT8 cells were treated with Sec C and cultured in 6-well low-adhesion culture plates, and then the spheres (diameter ≥ 60 μm) were counted. Scale bar, 500 μm. (**F**) Representative images of the wound from S100A8-knockdown HCT8 cells were recorded by microscope. Scale bar, 200 μm. (**G**) The Western blotting of S100A8-knockdown HCT8 and HT29 cells for stemness and EMT markers. Ctrl, the plasmid empty control. Sh or Si S100A8, the downregulated plasmid group. The data are shown as mean ± SD (*n* = 3). * *p* < 0.05, ** *p* < 0.01, *** *p* < 0.001, and **** *p* < 0.0001. NS indicated that no significance was noted.

Given that overexpressed S100A8 reversed the effect of Sec C on colorectal tumor cells, we continued to test the influence of downregulated S100A8 in the Sec C inhibitory procedure. Using the same method as before, we constructed stable S100A8-knockdown HCT8 and HT29 cell lines (Figure 5A). To assess cell growth, the colony formation assay and soft agar assay were used to explore the function of downregulated S100A8 in the Sec C killing process. We found that stable HCT8 and HT29 cells lost their growth capacity compared with the control, and Sec C increased its lethal activity. Taking HCT8 as an example, after downregulating S100A8, the colony numbers decreased by 69%, 49%, and 26% at 0.1 μmol/L, 0.3 μmol/L, and 0.5 μmol/L Sec C, respectively. In addition, the stereoscopic colony number decreased by 50% at 1 μmol/L Sec C under soft agar conditions. The tests using HT29 cells also showed the same phenomenon (Figure 5B,C). What is more, DNA replication and synthesis were also influenced by the knockdown of S100A8. Approximately 68% and 57% of EdU-positive cells of the control group under 0.5 and 1 μmol/L Sec C remained to replicate and multiply on HCT8 cells, respectively, and HT29 cells revealed the same repression as the S100A8 decreased (Figure 5D and Figure S4A).

As for the self-renewal ability, when S100A8 was downregulated, the spheres were much smaller than those of the control, and the number decreased significantly. With the 0.5 μmol/L Sec C treatment on HCT8 cells, approximately 66% of the spheres of the control survived and approximately 58% were alive under 1 μmol/L Sec C when almost no normal spheres could be seen in the wells of view. Concurrently, the HT29 cells showed a similar repression status, as shown in the Supplementary Materials (Figure 5E and Figure S4B). In addition, the migration capacity was also affected by the shortage of S100A8. Almost a 62% obstruction was detected under 1 μmol/L Sec C compared with the control group for HCT8 cells. Along with HT29 cells, it was obvious that Sec C displayed a stronger effect on the movement of tumor cells after S100A8 was downregulated (Figure 5F and Figure S4C). At the same time, Western blotting showed us that knocked-down S100A8 changed the expression of EMT and stemness markers after Sec C treatment. This confirmed that the loss of S100A8 helped Sec C to deprive tumor cells of stemness traits (Figure 5G).

Combining all results for S100A8, we concluded that S100A8 positively participated in the proliferation, migration, sphere formation, and drug resistance of tumor cells. Meanwhile, Sec C suppressed the stemness traits by decreasing S100A8 expression to inhibit tumor cell progression. In addition, S100A8 activation weakened Sec C activity, while S100A8 deficiency enhanced the Sec C lethal effect, which might play a profound role in cancer treatments.

### 3.6. Activation of p38 Weakens the Lethal Effect of Sec C on Colorectal Tumor Cells

**Figure 6:**
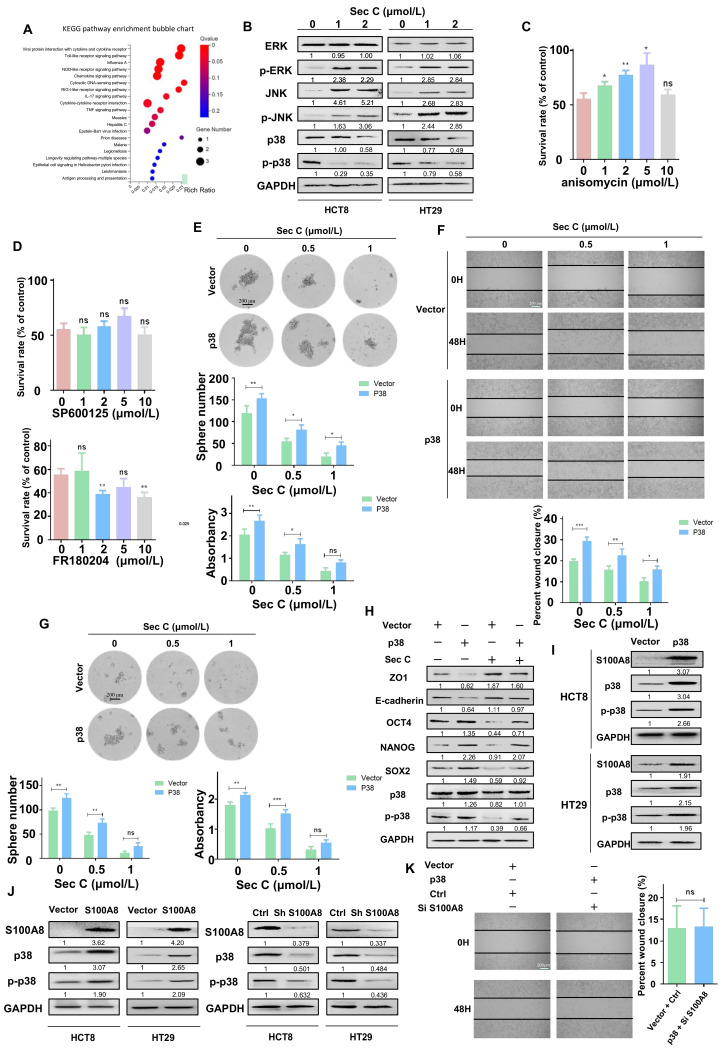
p38 weakens the effect of Sec C and interacts with S100A8. (**A**) The activated signaling pathways after S100A8 overexpression by RNA-seq analysis. (**B**) The changes of ERK, JNK, and p38 in Sec C-treated HCT8 and HT29 cells by Western blotting. (**C**) Anisomycin was mixed with Sec C to treat tumor cells in 96-well plates for 48 h, and the vitality of HCT8 cells were detected by MTT assay. (**D**) The survival rate of HCT8 cells treated by Sec C and SP600125 or FR180204 were detected by MTT assay. (**E**) The p38-overexpressing HCT8 cells were treated with Sec C for 24 h. Then, they were cultured in 6-well low-adhesion culture plates, and the spheres (diameter ≥ 60 μm) were counted. Scale bar, 200 μm. (**F**) The transfected HCT8 cells were seeded in 6-well plates and treated with Sec C. Representative images of the wound were recorded by microscope. Scale bar, 200 μm. (**G**) HCT8 cells with high p38 expression were cultured in serum-free medium, and then treated with Sec C for 24 h. The spheres (diameter ≥ 60 μm) were counted. Scale bar, 200 μm. (**H**) The proteins were extracted from transfected HCT8 cells after being treated with Sec C and immunoblotting was performed for stemness and EMT markers. (**I**) After p38 was overexpressed, S100A8 protein was detected in HCT8 and HT29 cells by Western blotting. (**J**) S100A8 was up- or downregulated in HCT8 and HT29 cells, respectively, and p38 was detected by Western blotting. (**K**) Representative images of the wound from HCT8 cells transfected by S100A8-downregulated and p38-upregulated plasmids were recorded by microscope. Scale bar, 200 μm. Vector, the plasmid empty vector. p38, the overexpressed plasmid group. The data are shown as mean ± SD (*n* = 3). * *p* < 0.05, ** *p* < 0.01, and *** *p* < 0.001. NS indicated that no significance was noted.

Next, we wanted to determine the pathways involved with S100A8 and Sec C. We extracted RNA from stable overexpressing S100A8 cells to carry out an RNA-seq analysis. After comparing with the previous RNA-seq analysis of Sec C-treated cells, we focused on IL17 signaling, especially the ERK, JNK, and p38 signal transduction pathways (Figure 4A and Figure 6A). From the Sec C-treated cells, we found that p38 was significantly decreased, while ERK and JNK were mainly activated (Figure 6B). We added anisomycin (p38 agonist), SP600125 (JNK inhibitor), and FR180204 (ERK inhibitor) to Sec C-treated cells, trying to weaken the Sec C killing effect. After 48 h of co-incubation, only anisomycin reversed the death of HCT8 cells, while SP600125 and FR180204 had no clear function. Consequently, we hypothesized that p38 might play an indispensable role in the Sec C killing process (Figure 6C,D and Figure S5A).

We enhanced the expression of p38 and examined the self-renewal ability as well as the migration capacity of HCT8 cells. It was apparent that the activation of p38 prompted tumor cells to move and grow into spheres, and the killing effect of Sec C was also weakened by p38 activation. An approximately 229% increase was observed for the 1 μmol/L Sec C-treated cells that finally grew into spheres, and a 150% increase was observed with the 0.5 μmol/L Sec C treatment. In terms of migration, after p38 was overexpressed, the closure rates were upregulated by 143% and 153% of the control at 0.5 and 1 μmol/L Sec C, respectively, indicating that p38 had an obvious reverse effect (Figure 6E,F). Meanwhile, spheres from upregulated p38 cells displayed a stronger resistance to Sec C. We found that the number of spheres increased by 153% and 224% at 0.5 and 1 μmol/L Sec C, respectively, and the shapes were larger with a higher density (Figure 6G). Western blotting confirmed that the expression of EMT and stemness markers were all influenced by p38 activation to undercut Sec C’s harmful effect on tumor cells (Figure 6H). All of these results fully demonstrated that p38 was significant in the Sec C inhibitory process and could hinder its anti-cancer activity.

Next, we detected the location of S100A8 and p38 in HCT8 cells, and the photo showed us that they had similar positions in the cells, implying that they might have a mutual regulatory effect (Figure S5B). We tested S100A8 expression in HCT8 and HT29 cells upon p38 activation and, as expected, p38 promoted S100A8 at the protein level (Figure 6I). Simultaneously, the result showed that S100A8 positively facilitated the protein level of p38; conversely, decreased S100A8 led to a low expression of p38 (Figure 6J). What is more, we raised p38 and knocked down S100A8 to test the migration of cells. Interestingly, there were no significant differences between the two groups (Figure 6K). Based on all of these results, we speculated that maybe S100A8 and p38 had a feed-forward loop, and some auxiliary regulatory pathways are involved in the balanced relationship between them.

### 3.7. Sec C Has Remarkable Anti-Tumor and CSCs Activity In Vivo

**Figure 7:**
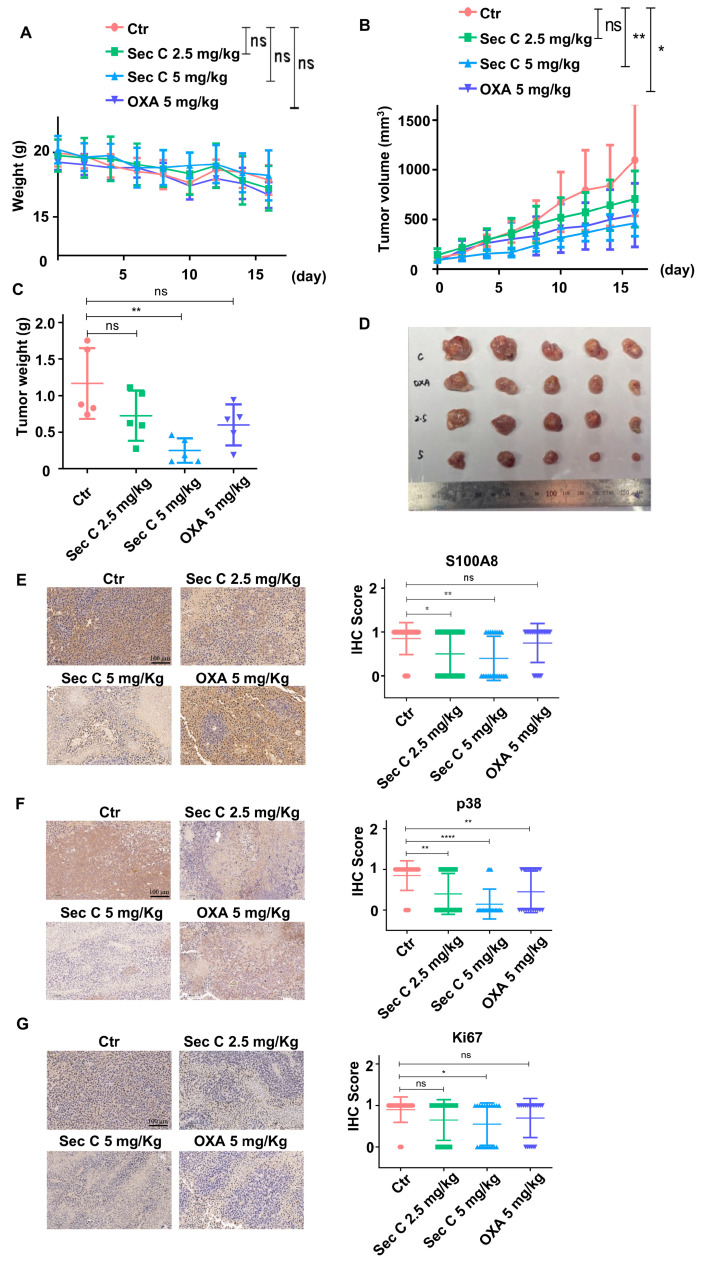
Sec C inhibits tumorigenesis in vivo. BALB/c nude mice were used to evaluate the anti-tumor activity of Sec C. After the mice were sacrificed, the variation curve of mouse weight (**A**), the variation curve of tumor volume (**B**), the weight (**C**), and the shapes (**D**) of tumors were all analyzed. The expression of S100A8 (**E**), p38 (**F**), and Ki67 (**G**) in the tumor tissue of BALB/c nude mice was detected by immunohistochemistry, and the IHC score was analyzed by ImageJ (Version 1.53). Scale bar, 100 μm. * *p* < 0.05, ** *p* < 0.01, and **** *p* < 0.0001. NS indicated that no significance was noted.

To verify the inhibitory effect of Sec C in vivo, we inoculated HCT8 cells subcutaneously in the nude mice, and intraperitoneally injected two doses of Sec C as well as the OXA control. After the mice were sacrificed, the variation curve of mouse weight (Figure 7A), the variation curve of tumor volume (Figure 7B), the final tumor weight (Figure 7C), and the shapes of the tumors (Figure 7D) were all analyzed. It was evident that, compared to the control group, the growth of colorectal tumors in the Sec C treatment group was slow and the average volume was small, indicating that the doses of 2.5 mg/Kg and 5 mg/Kg of Sec C inhibited the growth of colorectal tumor cells in nude mice. This effect was significantly better than that of the 5 mg/Kg dose in the OXA group.

HE staining confirmed that the main organs in the mice had good morphological characteristics and no obvious physiological changes. In other words, under the therapeutic dose in the experiment, Sec C did not exhibit significant toxic side effects (Figure S6A). We detected the expression of S100A8 and p38 in four groups of mouse tumors, and an IHC comparison showed that Sec C decreased the protein level of S100A8 and p38 in a dose-dependent manner. This was the same trend as that observed in vitro, while OXA played a smaller part in their expression (Figure 7E,F). Meantime, Ki67 was detected via IHC and the images showed that Sec C inhibited its expression even more effectively than OXA, indicating a prior anti-tumor activity of Sec C (Figure 7G). In summary, these results indicated that Sec C notably restrained the growth of tumors in vivo through S100A8 and p38, with a better effect than OXA and no obvious toxic side effects.

## 4. Discussion

Natural products are a huge source from which various drugs can be discovered. By studying potential compounds from plants, animals, and microorganisms, we have now applied many chemotherapeutic drugs to clinical treatments, such as streptomycin for tuberculosis, artemisinin for malaria, atropine for cardiac arrhythmia, and morphine for analgesia [57,58,59,60]. As for cancers, different natural derivatives have also made considerable contributions to human health, such as etoposide, doxorubicin, and daunorubicin, which lead us in a new direction for cancer prevention and treatment [61,62,63].

Our research team is continuously exploring natural products and have selected an active compound named Sec C from microbial fermentation. Since it was first reported in 1997, there have been few articles elucidating its roles and mechanisms in tumors. In our research, we found that Sec C effectively killed colorectal tumor cells with a significantly stronger activity than OXA both in vitro and in vivo. The results showed that Sec C played apparent inhibitory roles in CSCs, including affecting the proliferation, migration, self-renewal, and population of CSCs. Mechanistically, The Ca^2+^-binding protein S100A8 was most obviously downregulated in Sec C-treated colorectal tumor sphere cells, whose relationship with tumor stem cells has not been fully explained before [38,39]. Our subsequent experiments confirmed that S100A8 contributed to the stemness features, enhancing the resistance of colorectal tumor cells to Sec C and weakening the inhibitory effect of Sec C. At the same time, downregulated S100A8 led to the attenuation of colorectal tumor cells in stemness traits, which resulted in the vulnerability of tumor cells to Sec C and improved the inhibitory activity of Sec C. Furthermore, comparing the RNA-seq analysis results between Sec C-treated sphere cells and S100A8 overexpressed cells, we ultimately found that it was practical for the p38 signal pathway to promote the migration and sphere formation ability of tumor cells to reverse the inhibitory effect of Sec C on stemness traits. Interestingly, p38 and S100A8 were positively correlated and might have a mutual regulatory connection.

However, there are still some limitations in this study. Firstly, because of the lack of primary intestinal tumor cells or intestinal tumor organoids derived from patients, we were unable to directly detect the killing activity of Sec C and the function of S100A8 in a simulated human physiological environment [64,65,66]. Secondly, in our animal experiments, there was no significant toxicity of Sec C at the therapeutic doses. Based on the structure of Sec C, we speculate that it may trigger redox reactions, but further research is needed to determine the specific manifestation. Thirdly, more study should be conducted to deeply clarify the complex regulatory mechanisms regarding the relationship between p38 and S100A8. In the future, the above issues will be continuously tested with targeted approaches.

Overall, this study, for the first time, investigated the inhibitory effect of epitetrathiodioxopiperazine compound Sec C on colorectal tumor cells and found that Sec C exhibited strong repressive activity on CSCs and their stemness traits. What is more, it confirmed that S100A8 played a crucial role in the maintenance of the stemness features of tumor cells, accompanied by p38, which could also hinder the killing process of Sec C. This suggests that Sec C or its homologs have the potential to be developed as efficacious anti-cancer prodrugs after a series of structural renovations. Furthermore, S100A8 could be considered a novel target for drug design and a biomarker for the diagnosis and prognosis of cancer.

## 5. Conclusions

Sec C, a natural product derived from *Emericella* sp. via microbial fermentation, has obvious and rapid effects on killing colorectal tumor cells. Mechanistically, Sec C can inhibit the proliferation, migration, and self-renewal ability of tumor cells by downregulating Ca^2+^-binding protein S100A8. The p38-S100A8 feed-forward regulatory pathway can also actively participate in the maintenance of the stemness characteristics of colorectal tumor cells. Our research proves that Sec C is a promising prodrug for cancer treatment, and S100A8 can be a prospective target as well as biomarker for cancer screening and diagnosis.

## Data Availability

The data that support the findings of this study are available from the corresponding author upon reasonable request.

## Supplementary Materials

The following supporting information can be downloaded at: https://www.mdpi.com/article/10.3390/cells13070620/s1, Figure S1: (A) The IC_50_ of colorectal tumor cell lines HCT8, HT29, HCT116, RKO, HCT15 and CW2 under Sec C treatment for 48 h. (B) NCM460 was treated with Sec C for 48 h, and cell survival was detected with MTT assay. The dose-dependent curves were plotted via ImageJ (Version 1.53). (C) HCT15 and RKO cells were treated with Sec C and visible colonies were counted. (D) HCT116 and RKO cells were treated with Sec C for EdU assay. Scale bar, 300 μm. The data are shown as mean ± SD (*n* = 3). ** *p* < 0.01 and **** *p* < 0.0001. Figure S2: (A) RKO and HCT15 cells were treated with Sec C or OXA for 48 h, and cell viability was detected with SRB assay. The data are shown as mean ± SD (*n* = 3). ** *p* < 0.01 and **** *p* < 0.0001. NS indicated that no significance was noted. Figure S3: (A) The HT29 cells with high S100A8 expression were treated with Sec C and the DNA replication level was examined via EdU assay. Scale bar, 150 μm. (B) The stable HT29 cells were treated with Sec C and cultured in low-adhesion culture plates, then spheres (diameter ≥ 60 μm) were counted. Scale bar, 500 μm. (C) Representative images of the wound from S100A8-overexpressing HT29 cells were recorded via microscope. Scale bar, 200 μm. The data are shown as mean ± SD (*n* = 3). * *p* < 0.05, ** *p* < 0.01 and **** *p* < 0.0001. NS indicated that no significance was noted. Figure S4: (A) The HT29 cells with low S100A8 expression were treated with Sec C and the DNA replication level was examined via EdU assay. Scale bar, 75 μm. (B) The stable HT29 cells were treated with Sec C and cultured in low-adhesion culture plates, then spheres (diameter ≥ 60 μm) were counted. Scale bar, 500 μm. (C) Representative images of the wound from S100A8-knockdown HT29 cells were recorded via microscope. Scale bar, 200 μm. The data are shown as mean ± SD (*n* = 3). * *p* < 0.05, ** *p* < 0.01, *** *p* < 0.001, and **** *p* < 0.0001. NS indicated that no significance was noted. Figure S5: (A) The survival rate of HT29 cells after the co-incubation of Sec C combined with anisomycin, SP600125, and FR180204, separately, were tested via MTT assay at 48 h. (B) The locations of S100A8 (red) and p38 (green) were detected using immunofluorescence; scale bar, 50 μm. The data are shown as mean ± SD (*n* = 3). * *p* < 0.05, and NS indicated that no significance was noted. Figure S6: (A) Hematoxylin–eosin staining was conducted to detect the organ and tumor changes in BALB/c nude mice after receiving treatment with Sec C or OXA; scale bar, 50 μm or 100 μm.
