# Supplementary material for: The Natural Product Secoemestrin C Inhibits Colorectal Cancer Stem Cells via p38–S100A8 Feed-Forward Regulatory Loop
Source: Cells. 2024 Apr 3;13(7):620. doi: 10.3390/cells13070620 (PMC11011747; doi:10.3390/cells13070620)

Supplement figure S1

A

Cell lines	HCT8	HT29	HCT116	RKO	HCT15	CW2
IC_{50} (μmol/L)	0.7292	0.5731	1.161	0.3273	1.172	0.8632

B

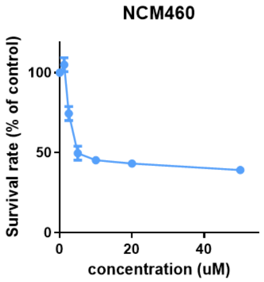

C

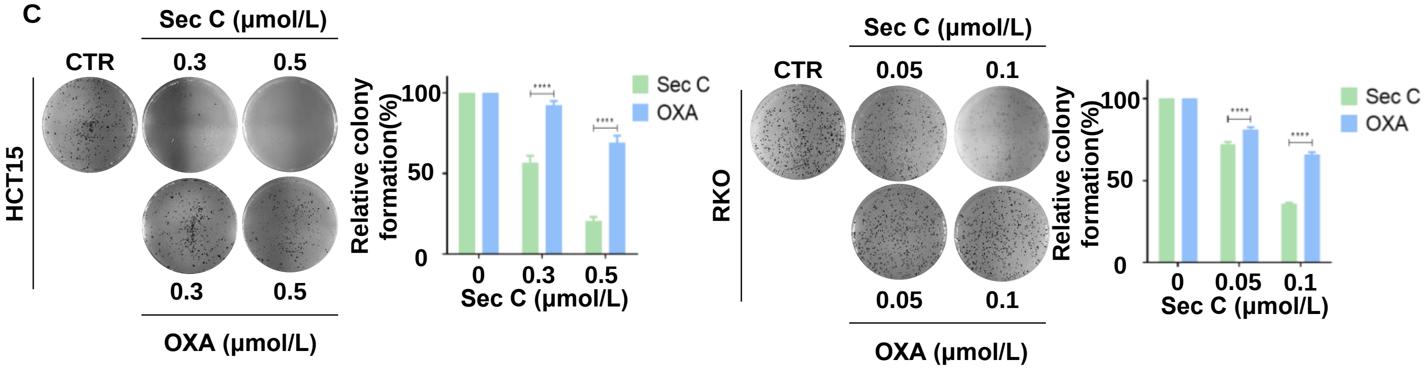

D

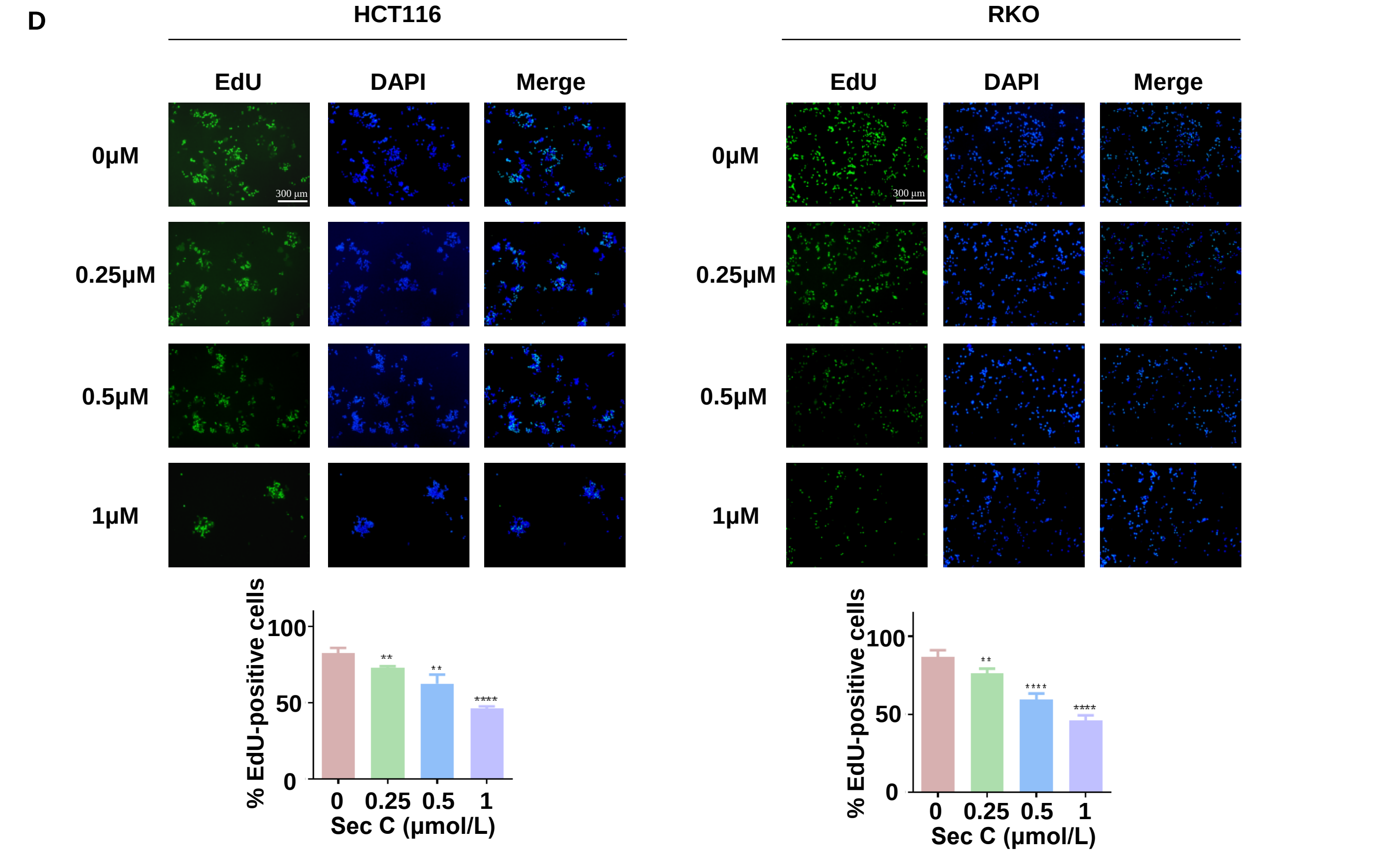

Supplement figure S2

A

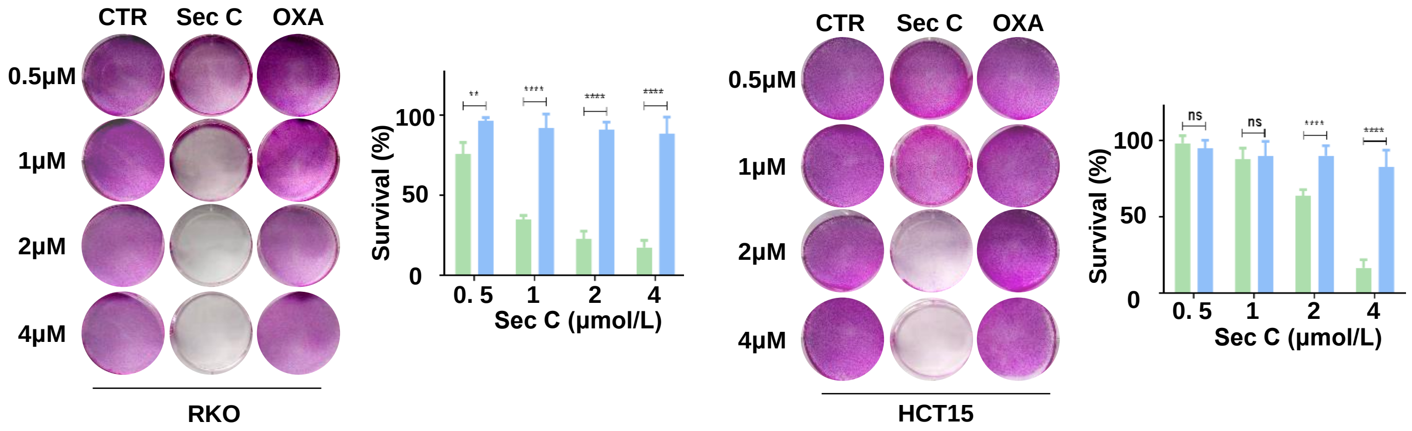

Supplement figure S3

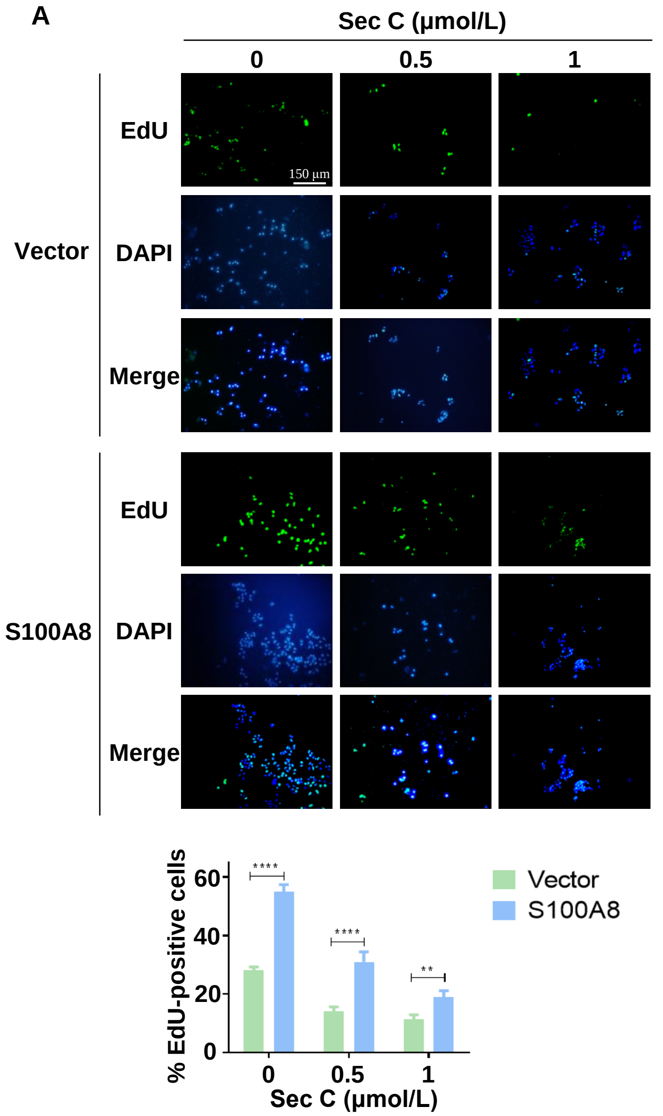

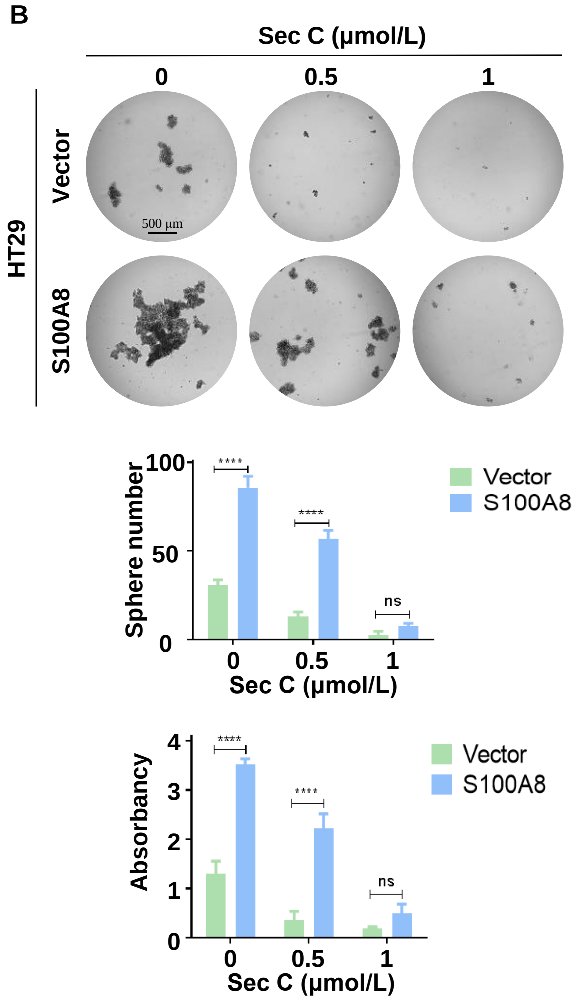

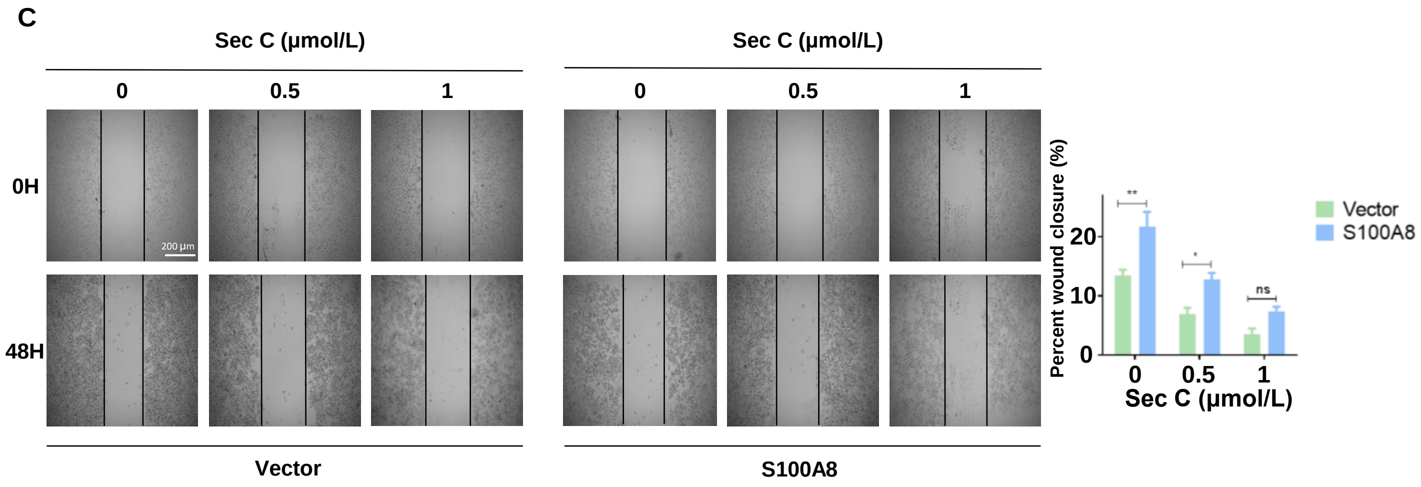

Supplement figure S4

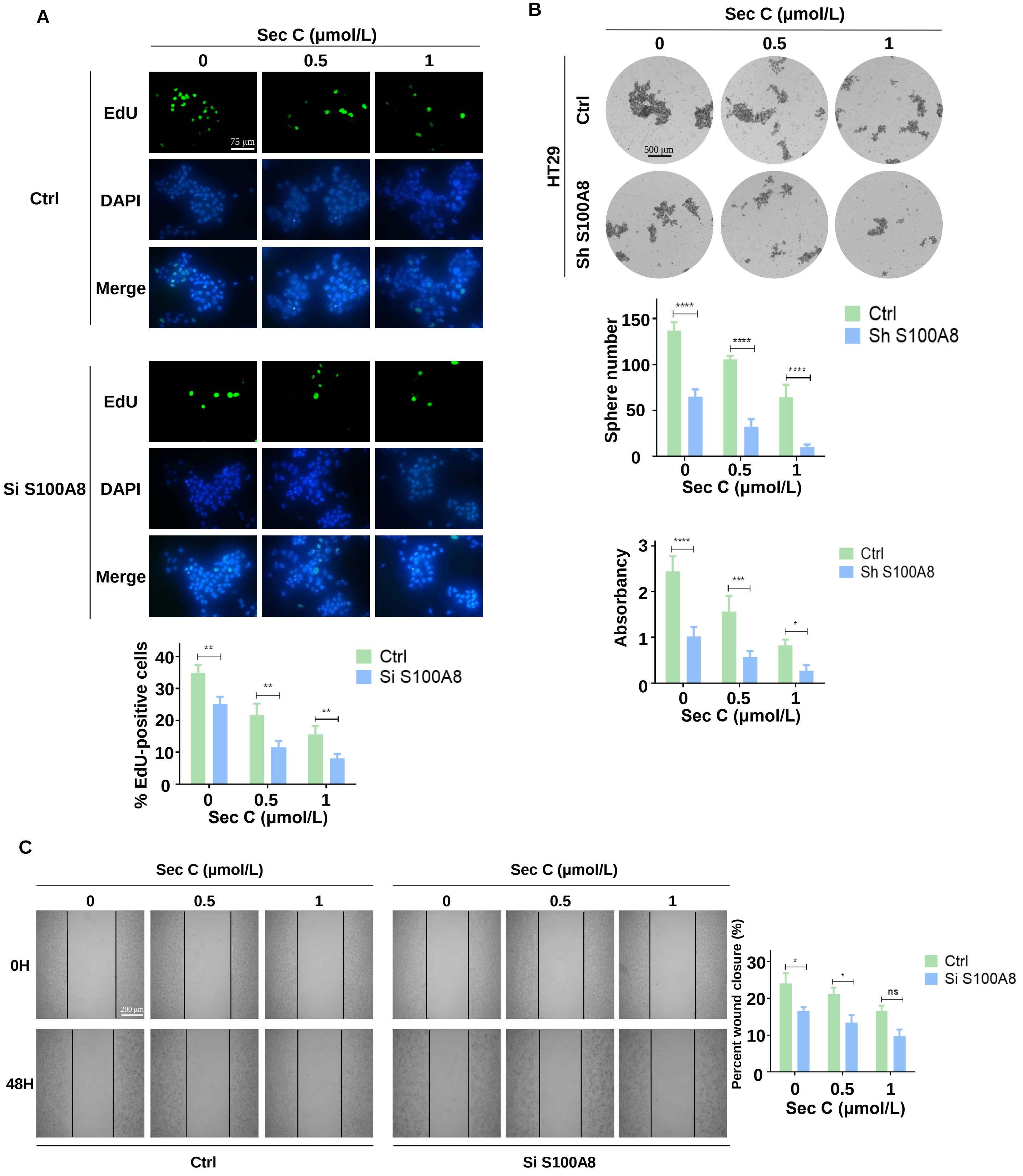

Supplement figure S5

A

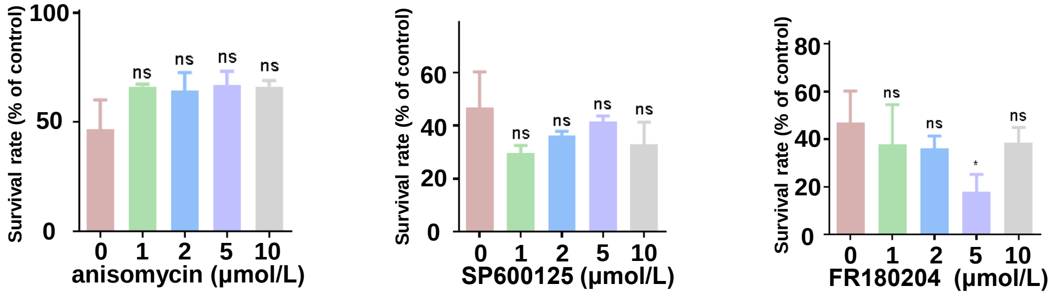

B

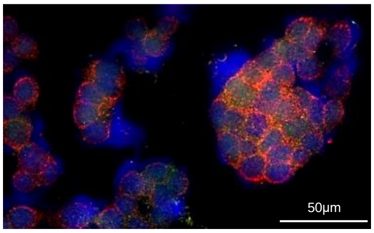

Supplement figure S6

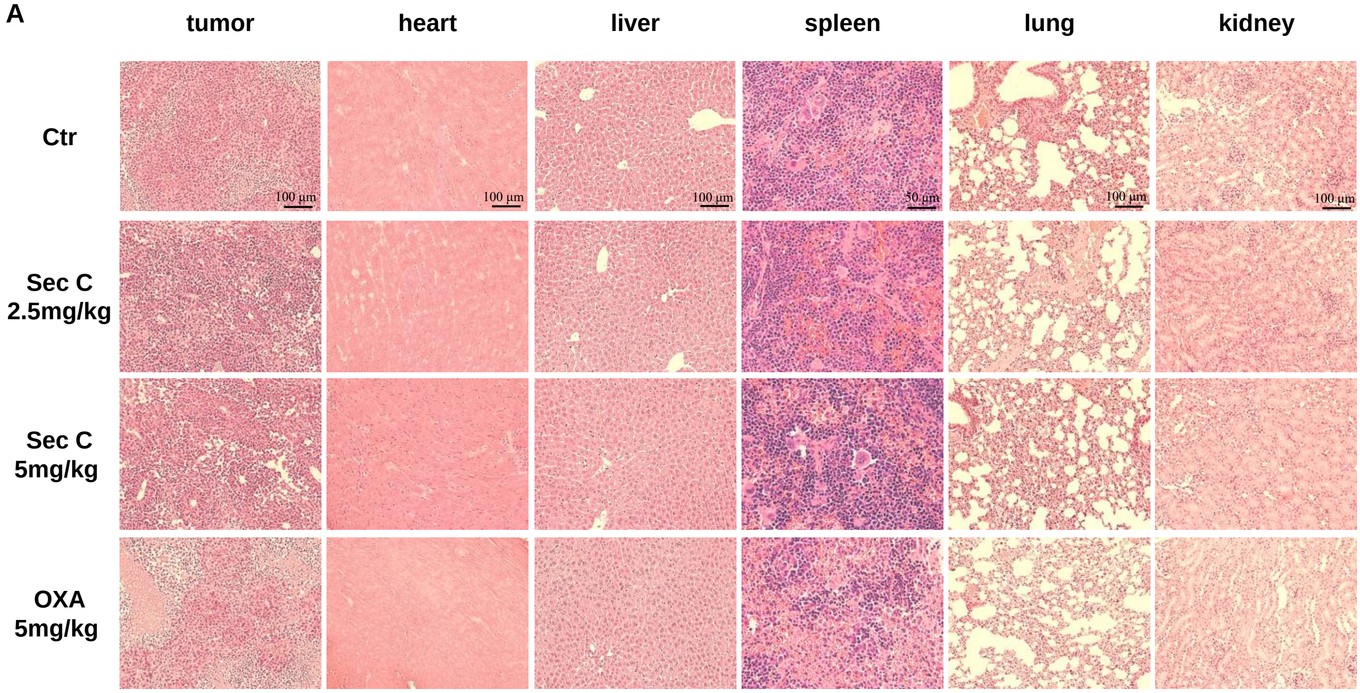

Supplement: Supplementary file 1 [file cells-13-00620-s001.zip › cells-2909770-supplementary.pdf]
